# Evaluation of immediate allergic reactions to dipyrone using dipyrone metabolites in basophil activation test

**DOI:** 10.1186/2045-7022-4-S3-P33

**Published:** 2014-07-18

**Authors:** Maria Isabel Montanez, Elena Garcia-Martin, Adriana Ariza, Maria Jose Torres, Jose AG Agundez, Cristobalina Mayorga, Inmaculada Doña, Natalia Blanca-Lopez, Maria A Zambonino, Inmaculada Andreu, Miguel Blanca

**Affiliations:** 1 Research Laboratory, IBIMA, Regional University Hospital of Malaga, UMA, Spain; 2 University of Extremadura, Department of Biochemistry & Molecular Biology, Spain; 3 IBIMA, Regional University Hospital of Malaga, UMA, Research Laboratory, Spain; 4 IBIMA, Regional University Hospital of Malaga, UMA, Allergy Unit, Spain; 5 University of Extremadura, Dept. of Pharmacology, Spain; 6 Infanta Leonor Hospital, Allergy Service, Spain; 7 La Fe Hospital, Unidad Mixta de Investigación IIS La Fe-UPV, Spain

## Background

Immediate selective hypersensitivity reactions to pyrazolones are thought to be mediated by specific-IgE antibodies. The diagnosis of selective reactions to pyrazolones is mainly based on the clinical history, skin testing and/or drug provocation test (DPT). Skin testing has low sensitivity and has potential risk of eliciting an anaphylactic response as well as DPT. Therefore the use of in vitro tests could be a safe and straightforward approach, although till now no well-validated test is available. Basophil activation test (BAT) has been proposed as a new in vitro diagnostic tool for different drug reactions although its sensibility is not optimal. The aim of this study was to analyze the potential usefulness of dipyrone metabolites to improve BAT sensitivity.

## Methods

The study included a group of 8 patients with a selective immediate hypersensitivity reaction to dipyrone, plus a group of 15 non-atopic controls tolerating dipyrone. BAT was done with dipyrone and four metabolites purified from human urine samples (4-methylamino antipyrine (MAA), 4-aminoantipyrine (AA), 4-acetylamino antipyrine (AAA) and 4-formylamino antipyrine (FAA). To confirm IgE-mediated basophil activation, the phosphatidylinositol 3-kinase (PI3-K) inhibitor wortmannin (WTM) was used in the BAT.

## Results

All patients were skin test positive in the immediate reading to dipyrone and had good tolerance to acetyl salicylic acid in drug provocation test. BAT results were positive in 2 patients to both dipyrone and MAA, in 2 patients to MAA alone and in 1 patient to AA alone. We confirmed that positive BAT results were an IgE-mediated response by using the WTM. All healthy controls showed negative BAT to dipyrone and metabolites.

## Conclusions

This study demonstrates the role of dipyrone metabolites in immediate reactions and supports the hypothesis that drug metabolites may underlie some allergic reactions. The additional use of dipyrone metabolites, besides the parent drug, increases BAT sensitivity, thus improving and refining the BAT-based diagnosis of selective reactions to dipyrone. These results deserve further studies using a higher number of patients and other dipyrone metabolites to gain deeper insight in the potential use of metabolite-based BAT as a target biomarker for allergic reactions to dipyrone.

